# Contrasting elevational patterns of soil and root-associated fungal communities highlight host-driven filtering in *Quercus wutaishansea* forests

**DOI:** 10.3389/fpls.2026.1825787

**Published:** 2026-04-17

**Authors:** Minyu Fu, Yulin Kang, Jianan Zhou, Lei Zhu, Wei Wang, Tong Lyu, Litao Lin

**Affiliations:** 1 State Key Laboratory of Environmental Criteria and Risk Assessment, Chinese Research Academy of Environmental Sciences, Beijing, China; 2 Institute of Environmental Information, Chinese Research Academy of Environmental Sciences, Beijing, China; 3 Department of Humanities and Social Sciences Research and Training, Fujian Institute of Education, Fuzhou, Fujian, China

**Keywords:** diversity, host filtering, *Quercus wutaishansea*, root-associated fungi, soil fungal community

## Abstract

The role of plant hosts in shaping root-associated microbial communities remains a central question in ecology, particularly in the face of changing environmental conditions. While considerable attention has been paid to soil microbial diversity, the interactive dynamics between plant individuals and soil microbial pool during root-associated fungi establishment across environmental gradients remain poorly understood. In this study, we explored the elevational variation in the diversity of soil and root-associated fungal communities of *Quercus wutaishansea* across ten elevational belts (1020 m-1770 m above sea level (asl)) on Dongling Mountain, Beijing, China. We found that root-associated fungal communities exhibited significantly increased alpha diversity with elevation (*P* < 0.05), whereas soil fungal communities showed no clear elevational trends (*P* > 0.05). Despite substantial variation in the soil fungal pool, the composition of root-associated fungal communities remained notably stable, suggesting a strong host filtering effect (*P* < 0.05). Compared with hump-shaped *Sim* and decreasing *Morisita β*-diversity of soil fungi as elevation increased (*P* < 0.05), the *β*-diversity of root-associated fungi did not exhibit a consistent elevational pattern nor mirror soil fungal *β*-diversity. These results suggest that, beyond environmental filtering, *Q. wutaishansea* plays an active role in shaping its root fungal community by selecting compatible fungal partners according to its physiological needs across altitudes. The findings reveal a significant and variable plant selectivity in the recruitment of microbiomes across different elevations, offering novel insights into plant-microbiome interactions within forest ecosystems in response to climate change.

## 1 Introduction

Host-symbiont specialization is widespread among plant species and plays an important role in structuring plant-fungal interactions (Bonfante and Genre, 2010; Kiers et al., 2011). Increasing evidence suggests that the assembly of root-associated fungal communities is not random but is shaped by multiple ecological processes, including host filtering, environmental selection, and dispersal limitation (Southworth et al., 2005; Davison et al., 2011; Tedersoo et al., 2020). Among these processes, host filtering is considered a key mechanism stabilizing mutualistic interactions, as plant identity and community composition can strongly influence fungal community assembly (Tedersoo et al., 2012; Hofmeister et al., 2014; Saitta et al., 2018). Consistent with this view, recent plant microbiome research highlights that plants can act as “microbiome engineers”, selectively recruiting beneficial microorganisms from the surrounding soil microbial pool (Zhang et al., 2024). Empirical studies across diverse ecosystems further demonstrate that host species identity significantly shapes the diversity and composition of root-associated fungal communities, even when plants grow under similar soil conditions (Sepp et al., 2019; Saitta et al., 2018). Large-scale forest studies also reveal that root-associated fungal assemblages often exhibit strong host dependence and may reflect the spatial distribution patterns of host plants within plant communities (Kuang et al., 2021).

However, recent studies have shown that both host traits and soil conditions can jointly influence the composition of root-associated fungal communities across forest ecosystems, although their relative importance may vary among environments and host species (Hogan et al., 2023; Du et al., 2023). As a crucial interface in the plant-soil system (Rillig and Mummey, 2006), root-fungal symbionts could be simultaneously restricted by the host plant and soil conditions (Entry et al., 2002). Elevational gradients provide natural laboratories for studying community assembly mechanisms because they encompass substantial changes in climate, soil properties, and vegetation over relatively short geographic distances. Environmental drivers are known to influence biodiversity patterns differently across functional groups and ecological strategies (Zhu et al., 2024), as demonstrated in studies of aquatic plant diversity across environmental gradients (Zhou et al., 2023). Temperature, moisture, and soil nutrient availability often vary predictably with elevation, leading to pronounced shifts in both plant and microbial communities (Delavaus et al., 2021). Numerous studies have reported significant changes in soil microbial diversity and composition along elevation gradients, including fungal communities (Yang et al., 2022; Peng et al., 2022; Lin et al., 2026b). Previous studies have examined fungal assemblages associated with single host species across elevation zones (Davey et al., 2013), and elevational partitioning of fungal communities has been reported (Jarvis et al., 2015). Nevertheless, it remains unclear whether turnover in root-associated fungal communities primarily reflects variation in soil fungal pools or context-dependent shifts in host filtering strength. Disentangling these alternative mechanisms requires explicitly partitioning the relative contributions of soil fungal composition and environmental variation to root fungal community structure.

Recent studies have shown that host regulation of symbiosis may vary across environmental contexts. Elevational variation can significantly reshape root-associated fungal diversity and community composition, often through shifts in soil nutrient availability and plant resource allocation strategies (Yang et al., 2021; Li et al., 2025). Empirical evidence indicates that host plants can reward higher-quality fungal symbionts through preferential carbon allocation (Werner and Kiers, 2015) and balancing carbon allocation among fungi for their efforts (Wang et al., 2017). Moreover, a microbial manipulation experiment showed diverse host promotion effects of microorganisms under different conditions (Wang et al., 2018), demonstrating that a plant species might have diverse preference expectation according to the specific circumstance. For common mycorrhizal networks, trees may show varying levels of preference/specialization (some species being more (or less) frequently connected than expected randomly) (Bahram et al., 2014). Together, these findings imply that host filtering intensity may not be fixed but instead vary with environmental contexts. Therefore, elucidating whether partner selection of plant species to be context-dependent or not is essential for the understanding of fungal community regulating mechanism across the elevation. However, direct evidence for such context-dependent filtering within a single plant species in natural ecosystems remains limited.

In this study, we investigated soil and root-associated fungal communities in ten *Q. wutaishansea* dominated forest belts spanning an elevational gradient from 1020 m-1770 m above sea level (asl). By comparing beta diversity patterns of soil and root fungal communities across elevation, we aimed to evaluate whether root fungal turnover can be fully explained by soil fungal pool variation. Therefore, we hypothesized that: (1) a lower fungal beta diversity in roots than that in soils, may indicate that significant preference of host plants in using soil fungal pools and (2) beta diversity of root fungal community was invariable across altitudinal transects or followed the same step with soil fungal beta diversity.

## 2 Materials and methods

### 2.1 Study site and sampling design

**Figure 1:**
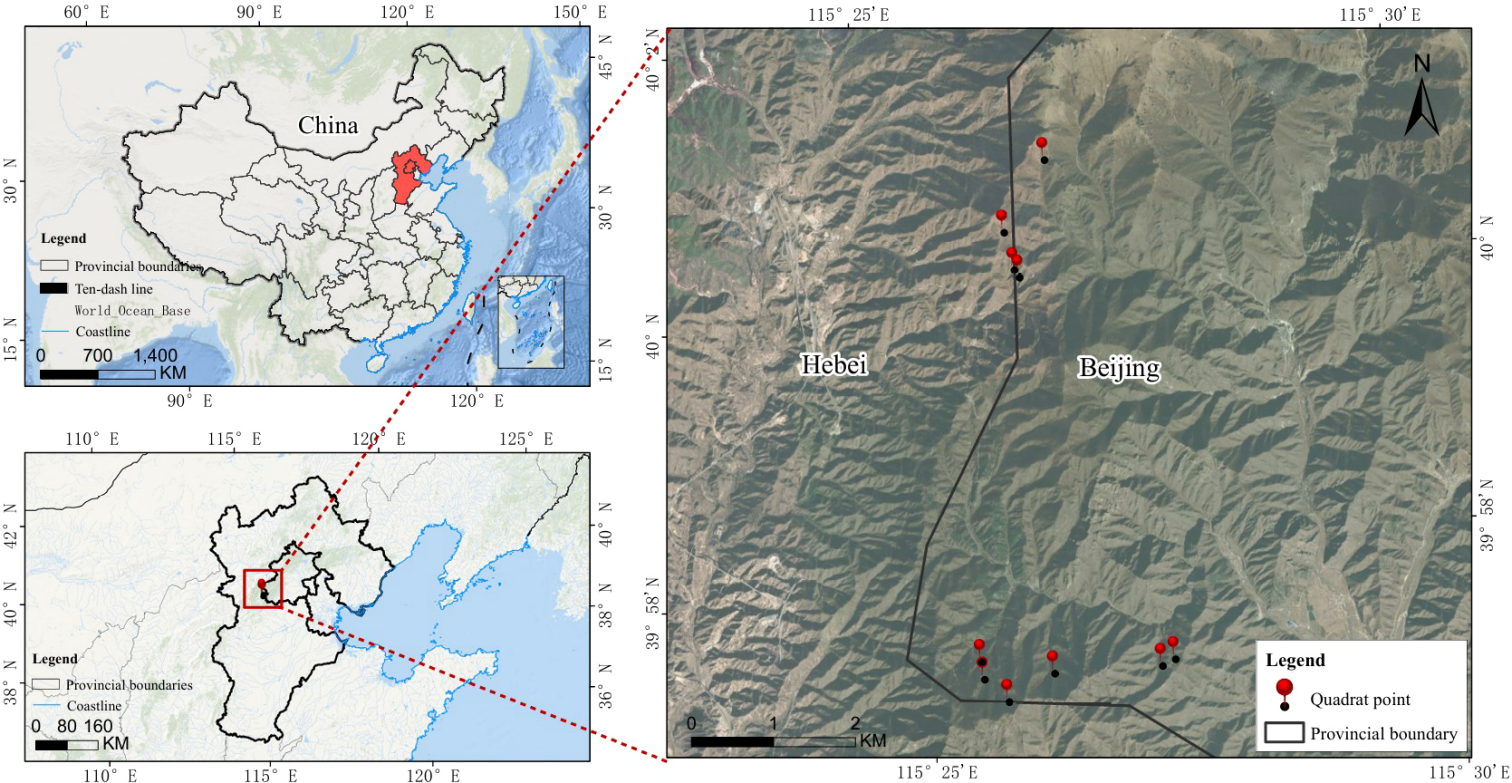
Sampling distribution of soil and root-associated fungi of *Q. wutaishansea* on Dongling Mountain, Beijing, China.

The study was conducted at the Beijing Forest Ecosystem Research Station of the Chinese Academy of Sciences (30°57′29″ N, 115°25′33″ E), located approximately 100 km northwest of Beijing city, China (**Figure 1**). The region has a typical warm temperate continental monsoon climate, with a mean annual precipitation of 500–600 mm and a mean annual temperature ranging from 5 to 10 °C. The dominant soil type in this area is brown soil. The forest represents an approximately 80-year-old secondary forest characterized by high structural heterogeneity. The canopy is mainly dominated by oak trees (*Q. wutaishansea*) with a few birches (*Betula* spp.), maples (*Acer mono.*), shrubs (e.g., *Prunus* spp.*, Vitex negundo* var. *hetertophylla*), and diverse herbaceous plants. To minimize heterogeneity among forest types, ten transects dominated by *Q. wutaishansea* were established along the western slope of the mountain. These transects collectively covered an elevational gradient from 1020 m to 1770 m asl, with each transect representing a distinct elevational segment.

In August 2013, along the ten western transects, ranging from 1020 m to 1770 m asl, three soil samples and three *Q*. *wutaishansea* roots samples were collected for each transect, resulting in a total of 30 soil samples and 30 root samples. For each *Q*. *wutaishansea* individual, roots in a 20 cm × 40 cm parallel and vertical slope 20 cm depth cross cube beside the tree were collected. Soil below the litter layer at a depth of 10 cm were then sampled, with a mixture of six holes from a 10 m × 10 m quadrat (sample tree being included). Roots within each sample were washed free from soil over a 1-mm sieve in running tap water. All fine roots (< 2 mm diameter) were cut into fragments c. 1 cm in length, and all the root tips from the root fragments were picked. The root samples were washed with sterilized distilled water and stored at -80 °C until DNA extraction. The fresh soil samples were thoroughly mixed, sieved through a 2 mm sieve and divided into two sub-samples. One sub-sample was air-dried for physical and chemical analyses, and the other was kept at -80 °C for DNA extraction.

### 2.2 Soil characteristics analyses

Soil moisture (SM) was determined using the gravimetric method. Soil pH and soil electrical conductivity (EC) was measured at a soil to water ratio of 1:2.5 (w/v). Soil organic carbon (SOC) was determined using the potassium dichromate oxidation method (Walkley, 1947; Walkley and Black, 1934). Soil total nitrogen (TN) was measured with a C/N analyzer (Vario EL III; Elementar, Langenselbold, Hessen, Germany). Soil total phosphorus (TP) was measured using the Mo-Sb colorimetric method (Yuan and Lavkulich 1995). The C:N and C:P ratios were calculated based on measured SOC, TN, and TP. Soil particles were classified into clay (0-2 μm), silt (2-50 μm) and sand (50-2,000 μm) using a Mastersizer 2000 Laser Diffraction Particle Analyzer (Malvern Instruments, UK).

### 2.3 DNA extraction and PCR amplification

Root samples were first pulverized in liquid nitrogen using a sterilized mortar and pestle. Total genomic DNA was extracted from 0.05 g of each root sample using a MOBIO PowerPlant DNA Isolation Kit (MO Bio Laboratories, USA) following the manufacturer’s protocol for maximum DNA yields. Soil DNA was extracted from 0.25 g of freeze-dried soil using PowerSoil DNA Isolation Kit (MO Bio Laboratories, USA). The quality and concentration of extracted DNA were assessed using a NanoDrop spectrophotometer (Thermo Fisher Scientific, USA) by measuring absorbance ratio at 260/280 nm and 260/230 nm. All DNA samples were stored at -80 °C until further analysis.

For roots samples, the internal transcribed spacer 1 region of the nuclear ribosomal RNA genes were amplified using primers ITS1F(5′-CTTGGTCATTTAGAGGAAGTAA-3′.) (Gardes et al., 1993) and ITS2(5′-GCTGCGTTCTTCATCGATGC-3′) (Smith and Peay, 2014) Whereas the internal transcribed spacer 2 regions of the nuclear ribosomal RNA genes were used for soil fungal amplification with primers ITS3 (5′-GCATCGATGAAGAACGCAGC-3′) and ITS4 (5′-TCCTCCGCTTATTGATATGC-3′) (White et al., 1990). PCR amplification was involved (1) Target region and primers: For roots, the fungal internal transcribed spacer 1 (ITS1) region was amplified using the modified ITS1F and ITS2 primers, combined with adapter and barcode sequences. (2) PCR reaction system: Amplification was conducted containing 12.5 μL Premix Taq polymerase, 0.5 μL of each primer (5 mmol L^-^¹), approximately 10 ng of template DNA, and sterile deionized water to the final volume. (3) Thermal cycling conditions: The PCR cycling conditions were as follows: an initial denaturation at 94 °C for 30 s, followed by 35 cycles of denaturation at 94 °C for 15 s, annealing at 58 °C for 15 s, and extension at 72 °C for 30 s, with a final extension at 72 °C for 5 min. During the PCR amplification, nuclease free water was included as a negative control to monitor potential contamination. (4) Product verification and downstream processing: PCR products were verified using 1% agarose gel electrophoresis and visualized under UV light. Each sample was amplified in triplicate to reduce PCR bias, and the resulting products were pooled. Amplicons were purified using the AxyPrep DNA Gel Extraction Kit (AXYGEN, USA) and subsequently sequenced on the Illumina MiSeq PE250 platform (Illumina, USA).

### 2.4 Bioinformatic analysis

Raw sequence data were processed using QIIME (Caporaso et al., 2010) and vsearch (Rognes et al., 2016). Paired-end reads were merged based on sequence overlaps. Sequences were discarded if they were shorter than 200 bp, contained ambiguous bases, exhibited an average quality score below 30 within a 50-bp sliding window, or showed more than one mismatch in the primer region.

Operational taxonomic units (OTUs) were clustered at 97% sequence similarity following the multistep procedure recommended by Nguyen et al. (in press). Initial clustering and chimera removal were performed using USEARCH, followed by OTU clustering using UCLUST. Representative sequences from each OTU were selected based on the most abundant sequence and taxonomically assigned using the UNITE database with the UCLUST classifier. To minimize potential artifacts caused by sequencing errors, OTUs that were not assigned to fungi or contained fewer than five reads were removed prior to downstream analyses (Lindahl et al., 2013). To eliminate the effects of different read numbers among the plots on the deduced EM fungal community composition, the OTU table was rarefied to the minimum sequencing depth across all samples using the single_rarefaction.py command in QIIME (Schloss, 2009).

### 2.5 Statistical analysis

All statistical analyses were conducted using R version 3.3.3 (http://www.r-project.org/) except where otherwise noted. Diversity indices were calculated using the vegan package version 2.5–4 in R (Oksanen et al., 2013). Meta-community network was generated based on Spearman correlation coefficients among fungal operational taxonomic units (OTUs).

Hill diversity numbers were calculated using the following Equation 1:

(1) $$N_{\alpha }={\left(\sum_{i=1}^{S}P_{i}\right)}^{1/\left(1-\alpha \right)}$$

where N*α* is Hill diversity (Hill, 1973), and S is total number of species measured in the sample. $P_{i}$ is the proportional abundance of species *i* and *α* is a scale parameter. Some Hill numbers are the number of species with *α* = 0, Shannon diversity with *α* = 1, inverse Simpson with *α* = 2, diversity of rare species in the community with *α* = -1 or -2 (Tothmeresz, 1995; Zhang et al., 2006).

Beta diversity based on presence-absence data was calculated using the Simpson dissimilarity index Equation 2:

(2) $$sim=\frac{min(b,\;c)}{min(b,\;c)+a}$$

where sim is beta diversity considering for binary data (Simpson, 1949; Koleff et al., 2003). *a* is the number of species shared between two sites and *b, c* is the numbers of unique species (not shared) between two sites.

Beta diversity based on abundance data was calculated using the Morisita index Equation 3–5:

(3) $$morisita=\frac{1-2\sum_{i}(x_{ij}x_{ik})}{(\lambda_{j}+\lambda_{k})N_{j}N_{k}}$$

(4) $$\lambda_{j}=\frac{\sum x_{ij}(x_{ij}-1)}{\sum x_{ij}\sum (x_{ij}-1)}$$

(5) $$\lambda_{\text{k}}=\frac{\sum x_{ik}(x_{ik}-1)}{\sum ik\sum (x_{ik}-1)}$$

Where *morisita* is beta diversity considering for quantitative data (Morisita, 1959). $x_{ij}$ is individual counts of species *i* in *j* community, and $x_{ik}$ is individual counts of species *i* in community *k*. $N_{j}$, $N_{k}\;$ are total individual counts of all species in community *j* and *k.*

## 3 Results

### 3.1 Basic property of soil and fungi along the elevational gradient

Along the elevational gradient, soil moisture (SM) exhibited a mean value of 0.375 v v^−1^ and pH of 6.483, ranges from 0.227 to 0.606 v v^−1^ and from 5.440 to 7.520, respectively (**Supplementary Figure 1**). Specifically, SM (v/v) varied moderately among elevational belts, with higher values observed in B9 and B7 belts. Soil pH was relatively stable and ranged between 6.0 and 7.5. SOC, TN, and C:N exhibited an increasing trend with increasing elevation, with SOC and TN markedly higher in the B9 belt compared to others belts. Whereas, higher sandy content (%) were observed in mid-elevation B4 and B5 belts than others. The elevational gradient exhibited a EC of 204.8 [3.2, 305.0] dS/cm, TP of 614.4 [534.0,845.2] mg/kg, C:N of 13.84 [11.10, 16.75], and C:P of 68.47 [57.24, 100.91] (**Supplementary Figure 1**).

**Figure 2:**
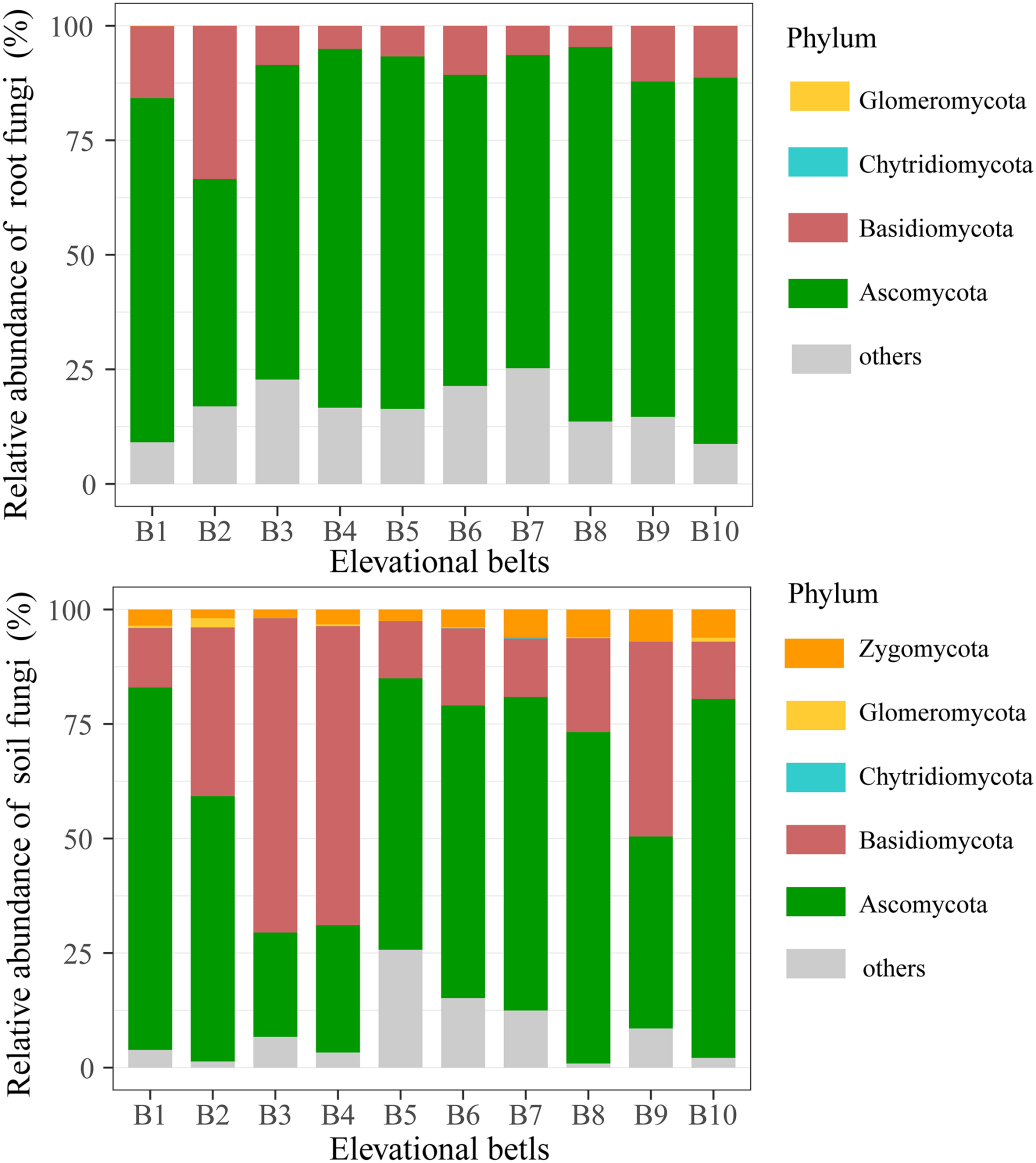
Taxonomic composition of root-associated and soil fungal communities.

The relative abundance of major phyla of soil and root fungi was quantified across the elevational gradient (B1-B10), with Ascomycota and Basidiomycota being the dominant taxa in both soil and root-associated fungal communities across all elevations (**Figure 2**). Specifically, Ascomycota consistently and Basidiomycota in soil communities represented the largest groups, with a relative abundance of 61.59% [22.83% (B3), 79.15% (B1)] and 18.65% [12.45% (B10), 68.58% (B3)], respectively. Compared with soil fungi, Ascomycota of root fungi were further promoted during colonization, with a relative abundance of 74.19% [49.64% (B1), 81.78% (T9)], whereas Basidiomycota was inhibited, with a relative abundance of 9.60% [4.61% (B8), 33.42% (B2)]. Phyla Glomeromycota and Chytridiomycota were detected at low but variable abundances across the gradient.

### 3.2 Fungal *α* diversity along the elevational gradient

**Figure 3:**
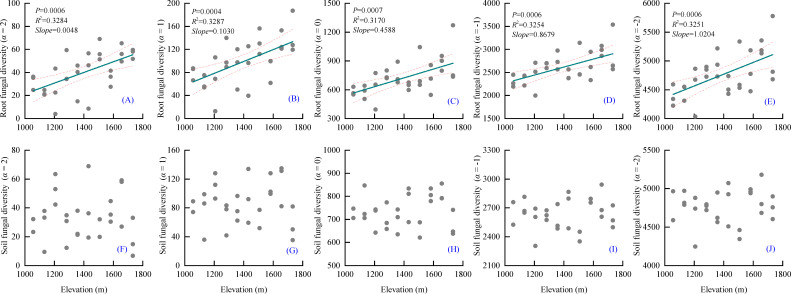
Variations of root associated and soil fungal diversity along the elevational gradient. **(A-E)**, root-associated; **(F-J)**, soil fungi; α = 2, α = 1, represent diversity of dominant groups in the community; α = 0, equal to community species richness; α = -1, α = -2, represent diversity of rare species in the community.

Alpha diversity showed distinct differences between soil and root-associated fungal communities along the elevational gradient (**Figure 3**). For root-associated fungal communities, with increasing elevation, root-associated alpha diversity showed a significantly increasing trend, showing similar patterns across diversity orders. Dominant taxa (*α* = 1 and *α* = 2) increased significantly with elevation, regression analyses showing a positive relationship. Species richness (*α* = 0) also increased significantly along the elevational gradient. Diversity indices that rare taxa (*α* = -1, -2) exhibited comparable patterns, with significant positive relationships between elevation and diversity (**Figure 3A-E**, all *P* < 0.05).

In contrast, soil fungal communities showed no significant elevational trends in alpha diversity. For soil fungal communities, regression analyses revealed no significant relationships in soil fungal alpha diversity for either dominant taxa (*α* = 1 and *α* = 2) or rare taxa (*α* = -1, -2) (**Figure 3F-J**; all *P* > 0.05). The scatter distribution of soil fungal diversity values appeared relatively stable across the elevational gradient.

### 3.3 Comparison of *β* diversity between soil and root-associated fungal communities

**Figure 4:**
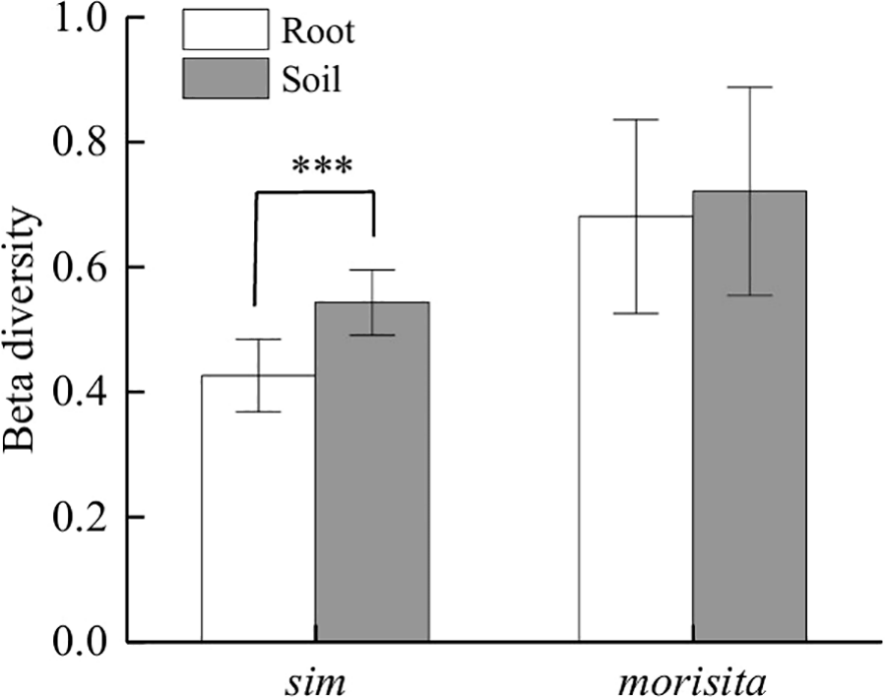
Values of soil and root-associated fungal beta diversity based on data of adjacent transacts. ***denotes a statistically extremely significant difference (P < 0.001).

The result showed that *β* diversity differed significantly between soil and root-associated fungal communities along the elevational gradient (**Figure 4**). Two complementary indices, one is the Simpson dissimilarity index, which only considers the presence or absence of species, and the other is Morisita index, which includes information on species abundance were used to characterize community composition turnover.

For the Simpson index, soil fungal communities exhibited significantly higher beta diversity than root-associated fungal communities (**Figure 4**, *P* < 0.001). The distribution of Simpson *β* diversity values indicated greater compositional differences between adjacent elevational transects for soil fungi compared with root-associated fungi. whereas root-associated fungal communities showed comparatively lower compositional dissimilarity along the elevational gradient. For the Morisita index, soil fungal communities also showed slightly higher *β* diversity values than root-associated fungal communities. However, the difference between soil and root fungal communities was less than that observed with the Simpson dissimilarity index and the difference in Morisita beta diversity between soil and root-associated fungal communities was not statistically significant.

To further evaluate the effects of soil and root-associated fungal communities and elevation on beta diversity, a two-way ANOVA was conducted. For the Simpson index, both treatment and elevation had significant effects on beta diversity (**Table 1**). Treatment explained a large proportion of variation (*P* < 0.001), indicating a difference in species turnover between soil and root-associated fungal communities. Elevation also had a significant influence (*P* < 0.001). In addition, the interaction between treatment and elevation was significant (*P* < 0.001), showed that the elevational response of *β*-diversity differed between soil and root-associated fungal communities.

**Table 1:** Two-way ANOVA of beta diversity of adjacent altitudinal transects to treatment and elevation.

Beta diversity	Source	*Df*	*F* value	*P* value
*Sim*	Treatment	1	253.31	0.0000
	Elevation	8	7.66	0.0000
	Treatment ×Elevation	8	4.048	0.0000
*Morisita*	Treatment	1	1.69	0.1960
	Elevation	8	2.71	0.0090
	Treatment ×Elevation	8	3.36	0.0020

For the Morisita index, elevation (*P* = 0.009) and the interaction between treatment and elevation (*P* = 0.002) were significant, whereas the main treatment effect was not significant (*P* = 0.196). These results show that abundance-weighted beta diversity varied along the elevational gradient and that the elevational responses differed between soil and root-associated fungal communities.

### 3.4 Elevational patterns of fungal *β*-diversity between adjacent transects

**Figure 5:**
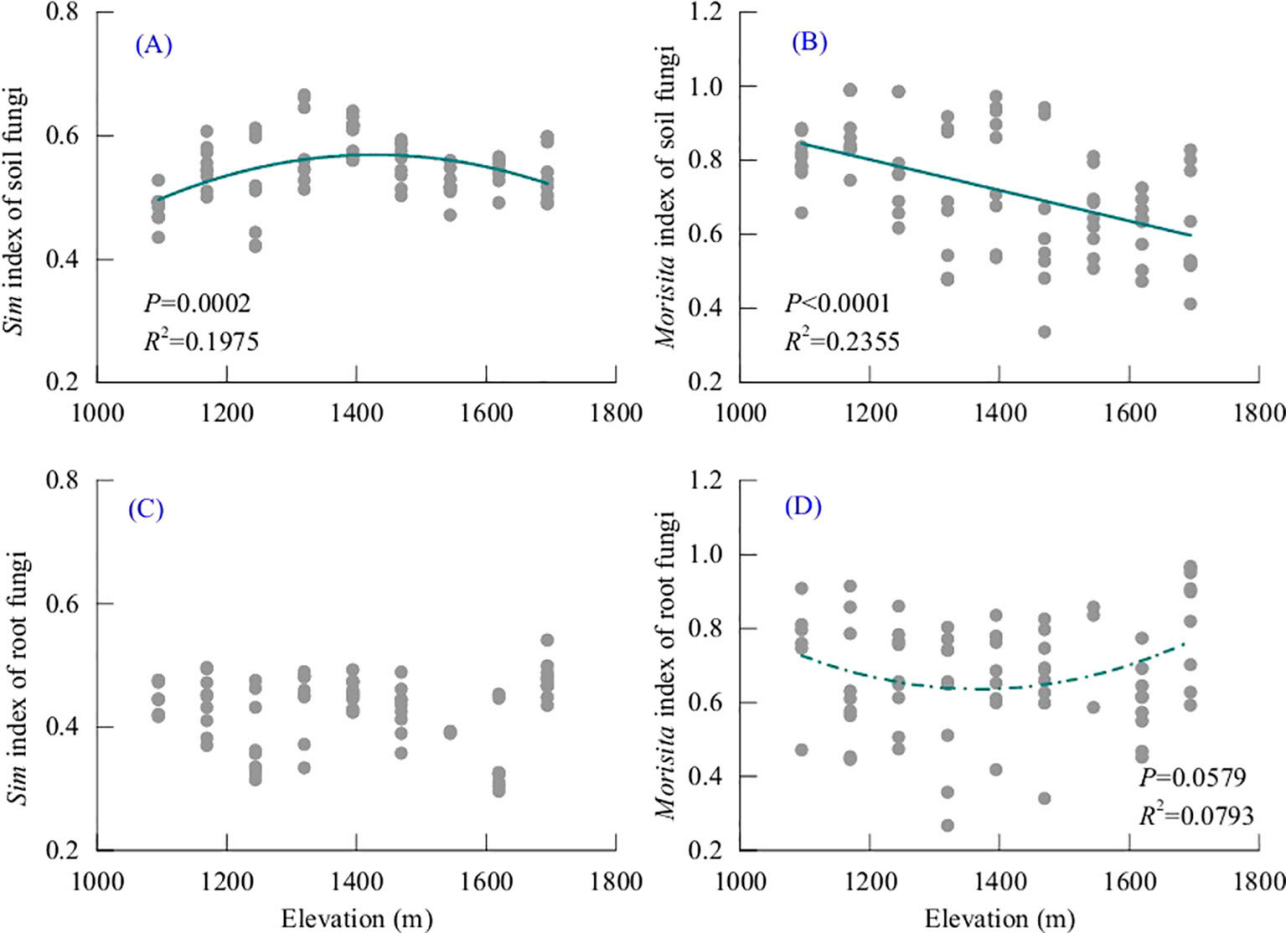
Changes in fungal beta diversity to increasing elevation based on data of adjacent transects. **(A, B)** are soil fungi; **(C, D)** are root-associated fungi.

The elevational patterns of beta diversity between adjacent transects further revealed differences between soil and root-associated fungal communities (**Figure 5**). For soil fungal, both Simpsson index and Morisita beta diversity showed significant relationships with elevation. Simpson beta diversity varied significantly along the elevational gradient (*P* = 0.0002) (**Figure 5A**). Beta diversity increased slightly from lower elevations to mid-elevations and then declined toward higher elevations, Morisita beta diversity of soil fungal communities also showed a significant elevational trend (*P* < 0.0001) (**Figure 5B**). In contrast to the Simpson pattern, Morisita beta diversity gradually decreased with increasing elevation.

Root-associated fungal beta diversity showed weaker or non-significant elevational relationships, particularly for Simpson *β-*diversity (**Figures 5C, D**). Simpson beta diversity showed no clear directional relationship with elevation (**Figure 5C**). Values were scattered across the elevational gradient and did not show consistent increasing or decreasing trends. For the Morisita index, (**Figure 5D**), *β* diversity of root-associated fungal showed a weaker elevational relationship compared with soil fungi (**Figure 5D**). The curve shows that *β* diversity gradually decreases with increasing elevation, is lowest at mid-elevations and then increases again.

### 3.5 Network structure of soil and root-associated fungal meta-communities

To further characterize the structural organization of fungal communities, meta-community network analyses were conducted for soil and root-associated fungi. The results revealed structural differences between the two fungal meta-community networks (**Table 2**). Root fungal networks exhibited higher connectance (0.2702) than soil fungal networks (0.1472). Similarly, the cluster coefficient was greater in root fungal networks (0.8827) than in soil fungi (0.6913). Root fungal networks also showed higher generality (13.06) compared with soil fungal networks (11.96). In contrast, soil fungal networks exhibited higher H2′ values (0.3941) than root fungal networks (0.2801). Differences were also observed in network nestedness. The NODF nestedness index was higher in root-associated fungal networks (40.27) than in soil fungal networks (23.84). A similar pattern was observed for sample-level nestedness (NODFsamples), which was higher in root-associated fungal networks (55.70) than in soil fungal networks (45.35).

**Table 2:** Property compression between soil and root fungal meta-community networks.

Network	Connectance	Cluster coefficient	Togetherness	H2’	Generality	NODF	NODF_samples_
Soil fungi	0.1472	0.6913	0.0343	0.3941	11.96	23.8350	45.3494
Root fungi	0.2702	0.8827	0.0644	0.2801	13.06	40.2660	55.6953

## 4 Discussion

### 4.1 Significant host selection from soil fungal pool

Understanding how species assemble to form communities is central issues in ecology and contributes to identify processes that govern species occurrences (Whittaker and Levin, 1977). In this study, we compared soil fungal communities with root-associated fungal communities along an elevational gradient to disentangle the relative roles of environmental filtering and host-mediated selection. Our results provide consistent evidence that *Q. wutaishansea* exerts strong selective filtering on the soil fungal pool during root fungal community assembly.

Despite substantial variation in soil fungal community composition along the elevational gradient, root-associated fungal communities presented a relative stable species composition pattern, with significantly lower Simpson beta diversity values than that in soils (**Figures 4**, **5**). This indicates that root fungal assemblages represent a filtered subset of the surrounding soil fungal pool, which is consistent with previous studies demonstrating that host plants can strongly regulate the composition of root-associated fungal communities (Kennedy et al., 2007; Pereira et al., 2012; Martinez-Garcia et al., 2015). Diverse selective preference among plant species during root fungal community establishment were confirmed by many researches (Newton and Haigh, 1998; Tedersoo et al., 2012; Bahram et al., 2014; Sepp et al., 2019), which also support great selective capacity of host plants from soil fungal pool.

Network analyses provided additional structural evidence for host filtering. The meta community network also showed a higher connectance level root-associated fungi than soil fungi, indicating higher co-shared fungi level of root fungi than that of soil fungi (**Table 2**). The more connected and clustered structure observed in root-associated networks suggests stronger host-mediated filtering and tighter co-occurrence patterns among root-associated taxa. In contrast, the relatively lower connectance and clustering in soil fungal networks reflect greater environmental heterogeneity and stochastic assembly processes. The reduced specialization (lower H2′) in root networks further indicates that a subset of compatible fungi repeatedly associated with the host across transects, reinforcing the role of host selection in shaping root fungal community structure. These results collectively suggest that *Q. wutaishansea* does not passively reflect variation in soil fungal communities but actively shapes the composition of its root-associated fungal microbiome through selective filtering processes. Similar patterns of increased connectivity and centralization within root-associated fungal networks have been reported in recent studies examining microbial interactions within plant root compartments, suggesting that plant roots create structured niches that promote stable microbial associations (Mo et al., 2025). 

### 4.2 Elevational patterns of *α*-diversity in soil and root-associated fungal communities

Elevational gradients are influencing microbial community composition through environmental filtering processes (Körner, 2007; Merges et al., 2018). Changes in vegetation composition and litter input along elevation gradients may further contribute to shifts in soil fungal communities (Newton and Haigh, 1998; van der Heijden et al., 2015; Gao et al., 2013).

In the present study, soil and root-associated fungal communities exhibited contrasting patterns of *α*-diversity along the elevational gradient. (**Figure 3**). Generally, certain percentage of fungi from soil pool was used in root fungal community, and similar alpha diversity variation pattern across elevational gradient should be observed between soil fungi and root fungi, for its random fungal selection existence in plant roots. However, in this study, no significant elevational alpha diversity pattern was observed in root fungal community. In contrast, root-associated fungal communities showed a significant increase in *α*-diversity with increasing elevation. This trend was consistent across diversity orders representing both dominant and rare taxa.

These results indicate that root-associated communities are not simply a random subset of the surrounding soil fungal pool. However, the strength of this filtering effect may vary among ecosystems, as some studies have reported that soil conditions can exert stronger influences than host traits on root fungal communities in certain forest systems (Hogan et al., 2023). If root colonization occurred through random recruitment from soil fungi, similar elevational patterns of *α*-diversity would be expected in both habitats. The contrasting diversity responses observed here instead suggest that host-mediated selection influences the composition of root fungal communities. Plants can influence fungal community composition by preferentially allocating carbon to beneficial fungal partners, thereby promoting the persistence of compatible symbionts while limiting less efficient or competitive taxa (Beiler et al., 2015; Zemunik et al., 2016; Cline et al., 2018).

Previous experimental studies have demonstrated that plants can preferentially allocate resources to more beneficial fungal partners (Kiers et al., 2011), thereby influencing community composition. Competitive interactions among fungal taxa within roots may further constrain coexistence, leading to reduced compositional variability relative to soils (Pickles et al., 2012). These researches likely contribute to the relatively stable composition of root-associated fungal communities observed in this study.

The increase in root-associated fungal *α*-diversity at higher elevations may therefore reflect adjustments in host-fungal associations under different environmental conditions. Elevational increases in root-associated fungal diversity have also been observed in other plant systems, suggesting that plants growing under harsher environmental conditions may rely more strongly on diverse microbial partners for nutrient acquisition and stress tolerance (Li et al., 2025). Environmental gradients can influence both plant physiological requirements and the composition of available microbial partners, potentially altering patterns of fungal recruitment within roots.

### 4.3 Environmental variation in host filtering along the elevational gradient

This study also found significant preference of *Q. wutaishansea* in choosing root-associated fungi from the soil fungi pools (**Figure 4**). Generally, plants species referred to cooperating with certain kinds of fungal taxon during the long periods of co-evolution (Gravel et al., 2006; Tedersoo et al., 2013; van der Heijden et al., 2015). However, how individual plants regulate fungal recruitment under changing environmental conditions remains poorly understood. Because soil fungal communities represent the immediate species pool from which root-associated fungi are recruited (Mahmoudi et al., 2019), shifts in environmental conditions are expected to influence both soil and root fungal communities through shared environmental drivers (Rincon et al., 2015). To eliminate interference of soil fungal pool replacement and verify weather host expectation variation impact on root fungal community or not, a comparison between soil beta diversity and root fungal diversity was used. Results showed, unlike the assumptions, the fungal community replacement was quite different between soil and root fungal fungi (**Figure 5**). For qualitative data, the *sim* beta diversity showed that root fungal community composition was much more stable at mid-domain elevation, despite relatively high turnover in soil fungal communities (**Figure 5**). This discrepancy suggests that stronger host filtering may occur at intermediate elevations, where plant roots selectively recruit a more consistent subset of fungal taxa from a diverse soil fungal pool. When fungal species abundance data was added, the soil fungal Simpson and Morisita beta diversity decreased with elevation increasing, yet Morisita beta diversity of root-associated fungi bottomed at mid-domain transactions (**Figure 5**). The inconsistent patterns between soil and root fungal communities indicated that host-mediated regulation of fungal recruitment may vary across environmental contexts. Rather than passively reflecting shifts in the soil fungal pool, plants may adjust fungal associations according to physiological requirements under changing environmental conditions.

The significant interactive effect of treatment and elevation on Simpson beta diversity also confirmed the significant fungal selective capacity of host individual under diverse environment conditions (**Table 1**), indicates that elevational effects on fungal turnover differed between soil and root habitats. This pattern suggests that host filtering intensity was not constant but varied across environmental contexts. Previous studies have also reported that fungal community assembly can vary depending on host characteristics and environmental context. For the same tree species of different succession stage forests, fungal community structure could be significantly different (Davison et al., 2011; Gao et al., 2015). Jonsson et al. (2016) experiment also showed divergent responses in *β*-diversity along the age gradient for litter fungi and toot fungi groups. Even in the same habitat, seedlings and mature trees showed distinctively different fungal community structures. A comparative study of different *Alnus rubra* stem density forest showed C-score value of fungal meta community network neutral or significantly lower than the random null models, yet number of significant correlation fungal OTUs being higher in low stem density areas (Kennedy et al., 2014). Across a steep precipitation gradient, C_4_ grass showed different preference in symbiotic fungal diversity and community composition (Giauque and Hawkes, 2013), yet the local environmental influence was unexcluded. These studies support the view that host-mediated regulation of fungal recruitment can vary under different ecological conditions.

In addition to host-driven processes, environmental variation may further modulate the strength of host filtering along the elevational gradient. The variation in soil physicochemical properties along the elevational gradient (**Supplementary Figure 1**) provides important environmental context for interpreting fungal community assembly. A robust body of evidence from diverse ecosystems underscores that soil factors are fundamental environmental filters shaping microbial communities. For instance, in similar mountain forest systems, soil pH, total phosphorus, and conductivity have been shown to be critical drivers of fungal diversity and composition, particularly for rare taxa (Lin et al., 2026a). This role extends beyond forests; in agricultural ecosystems, soil pH and nutrient content (e.g., SOM, TN, TP) consistently emerge as the primary determinants of microbial community structure for both bacteria and arbuscular mycorrhizal fungi (Yang et al., 2021; Zhu et al., 2024). These results suggest that environmental selection and host selection work together at different stages of community assembly. Soil conditions shape the regional fungal pool, whereas host plants actively regulate the composition of root-associated communities. This dual mechanism helps explain the observed effects between soil and root fungal diversity patterns along the elevational gradient.

Our results suggest that the strength of host filtering in *Q. wutaishansea* is environmentally contingent rather than fixed. Although soil fungal pools changed along with the elevational gradient, root fungal communities did not mirror these shifts proportionally, implying flexible host control over fungal assembly. However, the relative contributions of host regulation versus environmental filtering require further investigation using experimental approaches.

### 4.4 Ecological implications for plant–fungal interactions along environmental gradients

**Figure 6:**
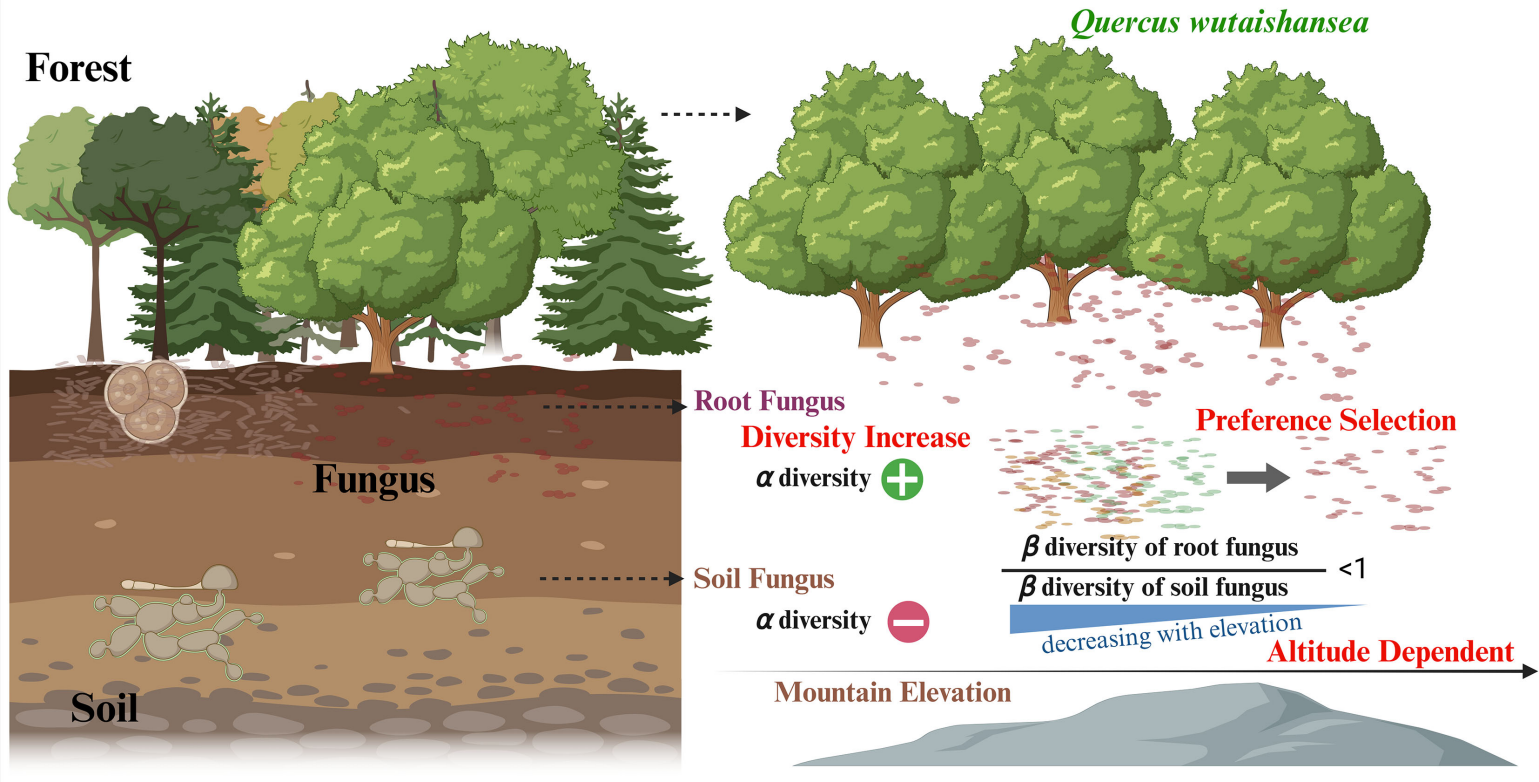
Host-driven filtering in root fungal assemblages with increasing elevation in *Q. wutaishansea* forests.

Plant-fungal symbioses play key roles in forest ecosystem functioning by influencing nutrient acquisition, soil processes, and plant productivity. As a dominant ectomycorrhizal tree species in temperate forests of northern China, *Q. wutaishansea* forms extensive associations with diverse fungal taxa that contribute to nutrient cycling and ecosystem stability. We compared the fungal communities of *Q. wutaishansea* root samples and soil samples along with ten elevational belts. The results showed that the root-associated fungal *α*-diversity increased significantly with elevation, whereas soil fungal diversity remained relatively stable along the gradient. Such divergence suggests that root fungal communities are not simply a subset of the surrounding soil fungal pool but are shaped by host-mediated recruitment processes, as conceptually illustrated in **Figure 6**.

Patterns of beta diversity further highlight this distinction. Soil fungal communities exhibited pronounced compositional turnover along the elevational gradient, reflecting strong environmental heterogeneity across sites. In contrast, root-associated fungal communities remained comparatively stable, with significantly lower beta diversity values. As summarized in **Figure 6**, this decoupling between soil and root fungal community dynamics indicates that *Q. wutaishansea* exerts a consistent filtering effect on its associated microbiome across elevations. This stability implies that *Q. wutaishansea* maintains a consistent set of compatible fungal partners despite substantial variation in the available soil fungal pool. Such selective recruitment likely reflects host preferences for specific fungal taxa capable of forming beneficial or functionally compatible associations.

These findings indicate that plant-microbe interactions are shaped not only by environmental filtering but also by active host selection. Host control over microbial recruitment may therefore represent an important ecological mechanism that enhances plant resilience to environmental stress by promoting beneficial microbial partnerships (Yue et al., 2024). The ability of plants to regulate their associated microbiomes may represent an important ecological mechanism facilitating plant adaptation to environmental gradients. Understanding these processes provides new insights into the role of plant-microbiome interactions in forest ecosystem resilience under ongoing climate change.

## 5 Conclusion

This study demonstrates that root-associated fungal communities of *Q. wutaishansea* are not simply a reflection of the soil fungal pool but are shaped by host filtering processes. Root fungal communities exhibited lower *β* diversity than soil fungal communities, indicating selective recruitment of fungal partners by the host plant. The strength of host filtering appeared to vary across the altitudinal gradient, suggesting that environmental context may influence plant-fungal interactions. This elevation-dependent filtering mechanism highlights the dynamic nature of plant control over symbiotic microbial communities. *Q. wutaishansea* plays a key role in shaping forest ecosystem structure and function, understanding the mechanisms governing fungal community assembly in *Q. wutaishansea* forests are particularly important for predicting how forest ecosystems may respond to environmental change. These findings contribute to a broader understanding of how environmental gradients and host regulation jointly shape microbial community assembly.

## Data Availability

The datasets presented in this study can be found in online repositories. The names of the repository/repositories and accession number(s) can be found below: https://www.ncbi.nlm.nih.gov/, PRJNA597700 https://www.ncbi.nlm.nih.gov/, PRJNA598051.

## Supplementary material

Supplementary Figure 1 Variation in soil physicochemical properties along the elevational gradient.
