# Supplementary figures and images for: Contrasting elevational patterns of soil and root-associated fungal communities highlight host-driven filtering in Quercus wutaishansea forests
Source: Front Plant Sci. 2026 Apr 17;17:1825787. doi: 10.3389/fpls.2026.1825787 (PMC13132827; doi:10.3389/fpls.2026.1825787)

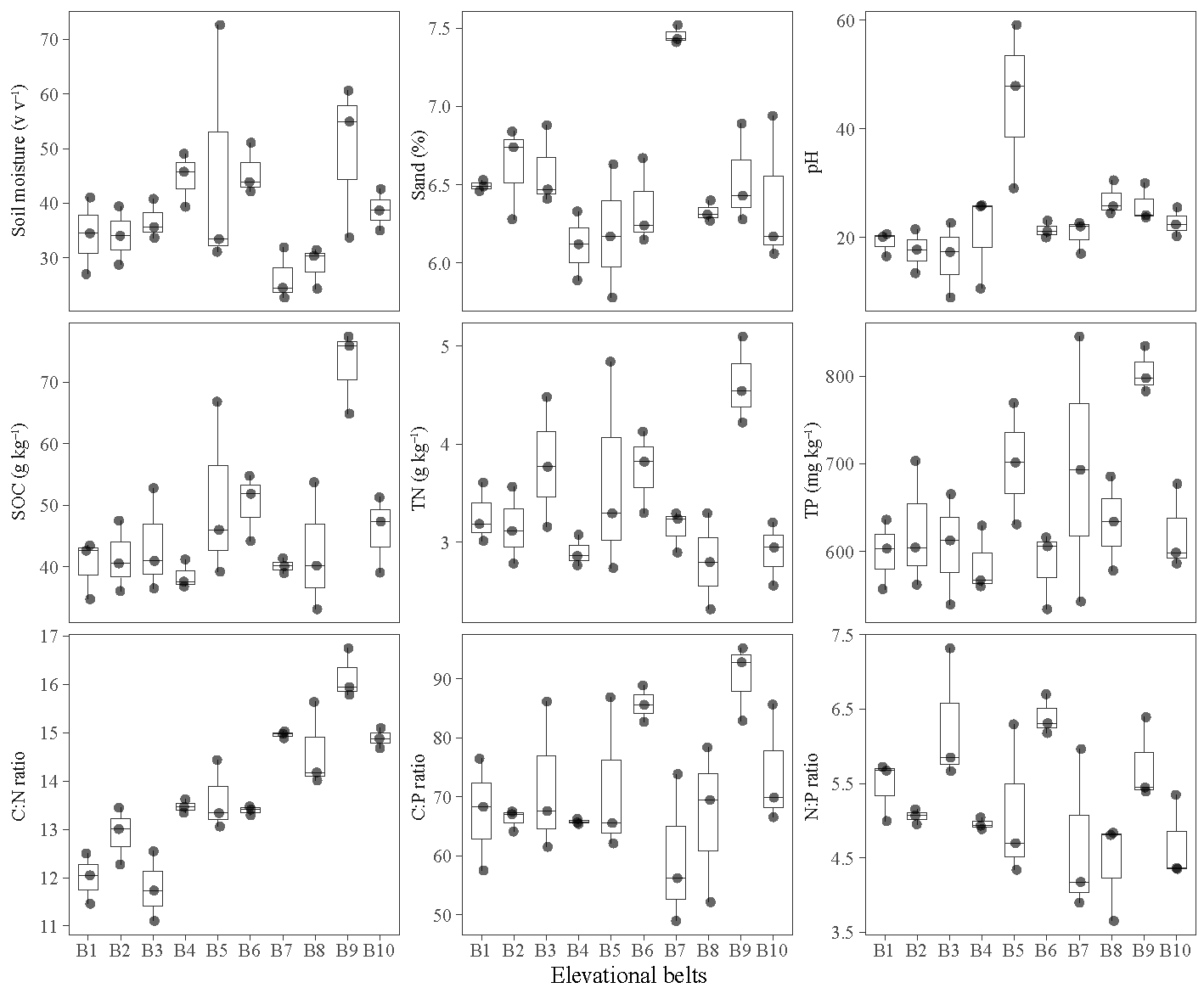

Supplement: Supplementary Figure 1 — Variation in soil physicochemical properties along the elevational gradient. [file Image1.png]
